# Development and Evaluation of a New Orthodontic Ligature: Frictional Force Analysis

**DOI:** 10.1055/s-0043-1768471

**Published:** 2023-06-13

**Authors:** Jaisson Cenci, Mauro Carlos Agner Busato, Veridiana Camilotti, Márcio José Mendonça

**Affiliations:** 1 Faculty of Dentistry, Federal University of Pelotas, Pelotas, Rio Grande do Sul, Brazil; 2 Western State University of Paraná, Dental School, Cascavel, PR, Brazil

**Keywords:** orthodontic brackets, orthodontic friction, orthodontic tooth movement, orthodontics

## Abstract

**Objective** To evaluate and compare the friction of different ligature modes used in orthodontics, and to propose a new ligature model for conventional brackets (“H low-friction orthodontic ligature).

**Materials and Methods** Samples were randomly divided into seven experimental groups: (1) resin H ligature (H3D), designed by the authors of this study and produced in a 3D printer, with conventional bracket; (2) metal H ligature (HFM), with conventional bracket; (3) passive self-ligating bracket (SLP); (4) “8” low-friction unconventional elastic (LT8), with conventional bracket; (5) loose conventional metal ligature (MLS), with conventional bracket; (6) conventional metal ligature fully tightened (MLT), with conventional bracket; (7) conventional elastic ligature (CEL), with conventional bracket—control. All samples were subjected to mechanical static friction testing using the EMIC DL 2000 universal testing machine.

**Statistical Analysis** To assess the normality requirement, the Shapiro–Wilk test was used, which showed a non-normal distribution for the means of the groups ( *p* < 0.05). Therefore, statistical tests were performed to assess the existence of statistically significant differences between the groups through the Kruskal–Wallis, followed by Dunn's test, pairwise comparison, *p* < 0.05.

**Results** The results obtained showed lower friction values for HFM (0.002 kgf), SLP (0.003 kgf), and LT8 (0.004 kgf)—these did not differ statistically from each other. These were followed by H3D (0.020 kgf), MLS (0.049 kgf), CEL (0.12 kgf), and, finally, MLT (0.21 kgf).

**Conclusion** The lowest friction value was found for the metal H ligature, similar to the self-ligating bracket and the “8” low-friction unconventional elastic. The resin H ligature presented intermediate friction values and the highest friction force was found for the MLT group.

## Introduction

Orthodontic treatment is based on tooth movement and this is performed by applying forces using brackets. 1 Traditional orthodontics works with the bracing of orthodontic wires, which can be connected to the slot of conventional brackets through different methods, resulting in different forces released to the teeth. 2 For this purpose, the most traditional and commonly used forms are metal ligatures and circular elastomeric ligatures. 1 2 3

Circular elastomeric ligatures have stood out among most orthodontists because they are more practical and efficient. 2 4 These models, known as conventional ligatures, apply a force that pushes the orthodontic wire against the base of the bracket slot, increasing the frictional forces, 5 hindering the sliding mechanics, reducing the speed of tooth movement at the beginning of the treatment, as well as to making it difficult to anchoring control in orthodontics traction mechanics. 6 7 In addition, of the total forces applied to orthodontic movement, 50% are dispersed only to overcome friction in the system. 8 9 10

On the other hand, the use of self-ligating brackets has become popular in recent years. 11 12 This system was developed in 1935, with the Russel Lock appliance, and consists of a preadjusted bracket that has a built-in mechanical device, usually on its buccal face, which serves as a cover, or precision lock to attach the orthodontic wire to the slot, eliminating the need for ligatures. 1 9 10 13 In this system, a tunnel is formed, and without contact, the orthodontic wire slides freely, reducing friction when compared with conventional ligatures, 5 7 10 13 14 15 16 17 as well as reducing the accumulation of dental biofilm when compared with the traditional system. 18

However, several studies have reported that the reduction of friction, provided by self-ligating brackets, is important in the initial stages of orthodontic treatment, leveling, and alignment, as well as for space closure and sliding mechanics. 17 22 The final stages of treatment, on the other hand, require greater frictional force, to obtain three-dimensional control of the position of the tooth. 6 16 19 Since in this stage the self-ligating brackets do not present a satisfactory result, the conventional brackets with conventional ligatures seem to present better three-dimensional control. 19 Another disadvantage of self-ligating brackets is the cost, as they are significantly more expensive than conventional brackets. 7 Thus, the ideal orthodontic system appears to be one in which the friction levels can be switched, depending on the treatment phase, without the need to change brackets or increase costs.

Therefore, a new ligature design was developed by the authors to simultaneously meet the ideal friction force requirements in the different stages of orthodontic treatment.

## Materials and Methods

### Study Design

#### Sample Size Calculation

The sample size calculation was made based on probability distributions of the F family, with repeated family design, with interaction within and between factors. The effect size was 0.15, 5% type 1 error and 95% power guaranteed a minimum of 105 sample units (specimens), with 15 samples per experimental group. The sample calculation was performed using the GPower software (GPower, version 3.1.9.2, University of Düsseldorf, Düsseldorf, Germany).

#### Ligature Design

**Fig. 1:**
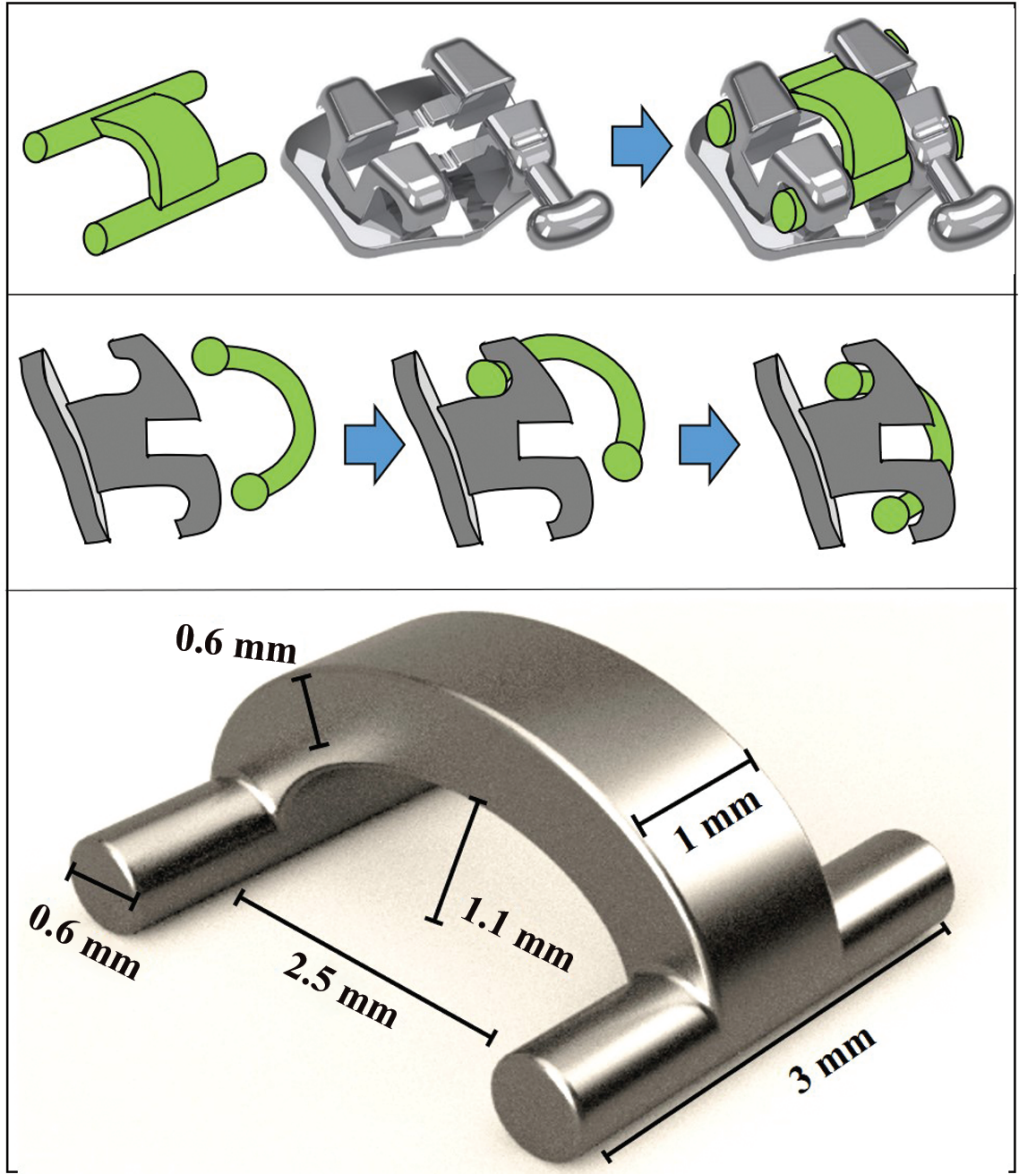
Layout and dimensions of the H ligature.

The “ **H** ”-shaped pieces, developed by the authors, were designed through trial and error, as follows:

- First, a draft drawing was made to measure a conventional metal bracket, more specifically measuring the spaces where the ligature would be inserted. The bracket used was a central incisor tooth, upper left, slot 0.022 inch × 0.028 inch from Morelli (Dental Morelli, São Paulo, SP, Brazil).
- Second, the first version of the H3D ligature was manufactured in resin for 3D printing (Resin DM-300; Markertech Labs, Tatuí, São Paulo, Brazil) through the 3D printer B9Creator v1.2, (B9Creations, Rapid City, South Dakota, United States). As this first version fits perfectly in the conventional metal bracket, no changes needed to be done, the H3D shape was defined, and more pieces were printed;
- For the H ligature metal version (HFM), an H3D ligature was used as a mold, to ensure the uniformity of the shape and dimensions of the H metal ligatures. HFM ligature was cast in metal (Ni-Cr alloy for Fitcast-V metalloceramics; Talmax, Curitiba, PR, Brazil), in a dental prosthesis laboratory, following a similar process and the same materials used to make metal pins or other dental appliances such as removable partial dentures. The layout of the part, as well as the design measures, is illustrated in Fig. 1 .

#### Experimental Groups

The friction strength of seven types of ligatures was assessed and is described in detail in Table 1 .

**Table 1:** Description of groups

Group	Description
H3D	H ligature, designed by the authors and produced in a 3D printer, installed in a conventional metallic bracket
HFM	Cast metal H ligature, made in a dental prosthesis laboratory using a model of the H3D ligation group, installed in a conventional metallic bracket.
SLP	Passive self-ligating bracket (SLP—self-ligating Roth; Morelli, São Paulo, Brazil)
LT8	Low friction elastic (elastic ligature in the shape of “8”; Tecnident, São Carlos, São Paulo, Brazil), installed in a conventional metallic bracket
MLS	Metal ligature 0.010 inch (ligature wire CrNi; Morelli), loose mooring, installed in a conventional metallic bracket
MLT	Metal ligature 0.010 inch (ligature wire CrNi; Morelli), strong mooring, installed in a conventional metallic bracket
CEL	Circular elastic ligature (Bengalinha elastic ligature; Morelli), installed in a conventional metallic bracket: control

**Fig. 2:**
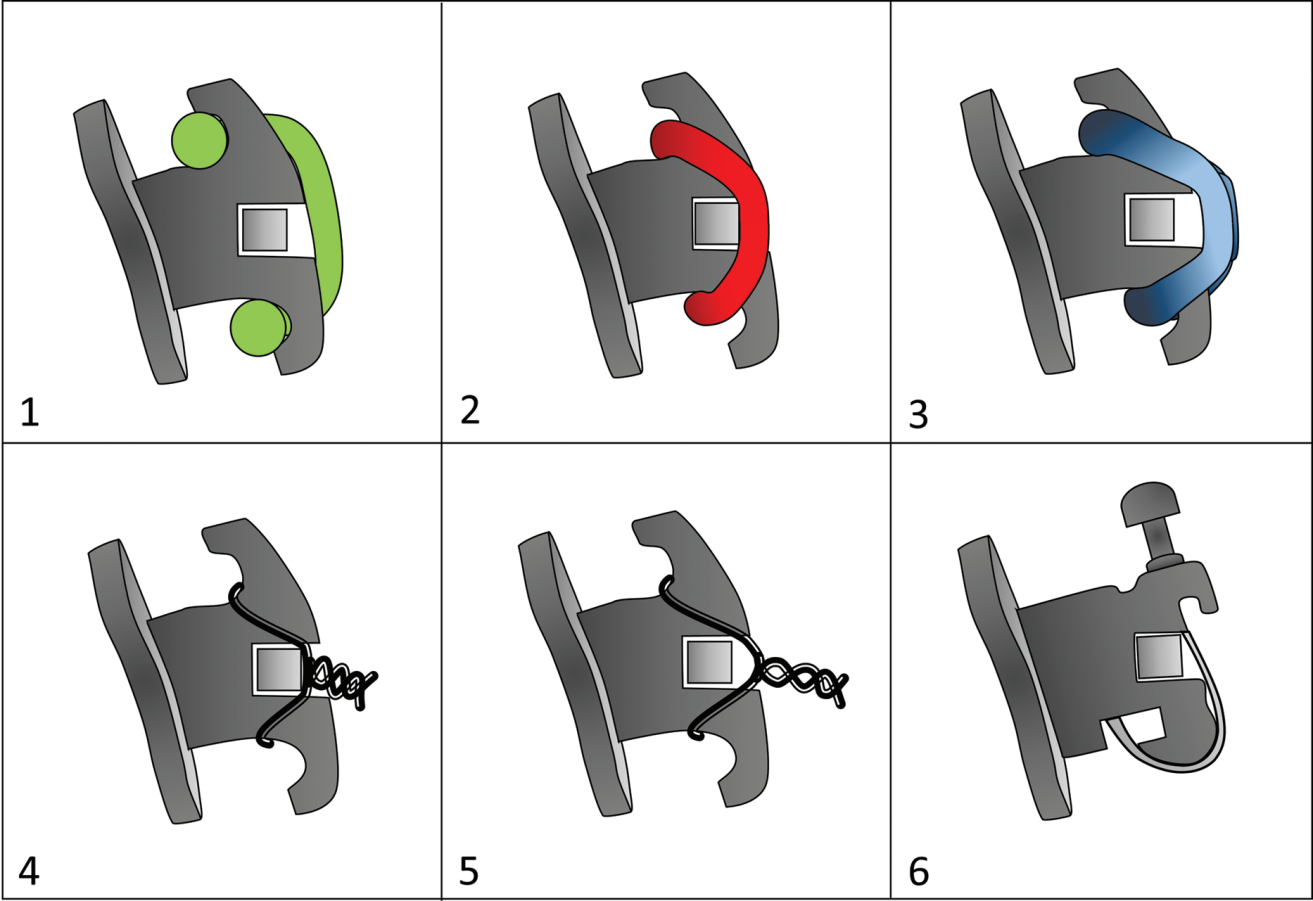
The comparative design of all ligature formats used in the study in lateral view. It is possible to observe in this schematic drawing that some types of ligatures do not touch the wire after being inserted, providing more freedom for the wire movement in the slot: (1) H ligature installed in the bracket (resin and metal H ligatures have exactly the same dimensions and formats); (2) conventional elastomeric ligature; (3) low friction elastic ligatures; (4) metal ligature tight; (5) metal ligature lose; (6) passive self-ligation bracket.

The visual differences between the types of ligatures are shown in Fig. 2 .

### Sample Preparation

The tests were performed using rectangular 0.019 inch × 0.025 inch stainless steel rod wires (Dental Morelli), 14 15 16 17 20 21 and conventional metallic brackets of the central incisor tooth, upper left, slot 0.022 inch × 0.028 inch, and Roth prescription (5-degree angle and 12-degree torque) in all groups, in the “Light model” for the conventional ones and in the SLP (self-ligating Roth) model for the self-ligating passive, both from the Dental Morelli company.

**Fig. 3:**
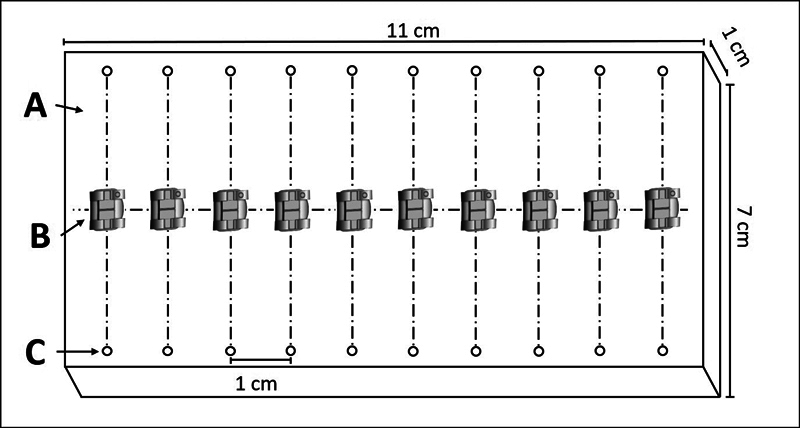
( **A** ) Rectangular acrylic device. ( **B** ) Orthodontic brackets. ( **C** ) Holes for calibration of the bracket bonding.

To standardize the samples, all the samples were prepared by the same operator. A rectangular acrylic device measuring 11 cm × 7 cm × 1 cm (W × D × H) was used, in which the brackets were bonded exactly in the center ( Fig. 3 ). This device was attached to the bottom of the universal testing machine.

#### Wire Confection

**Fig. 4:**
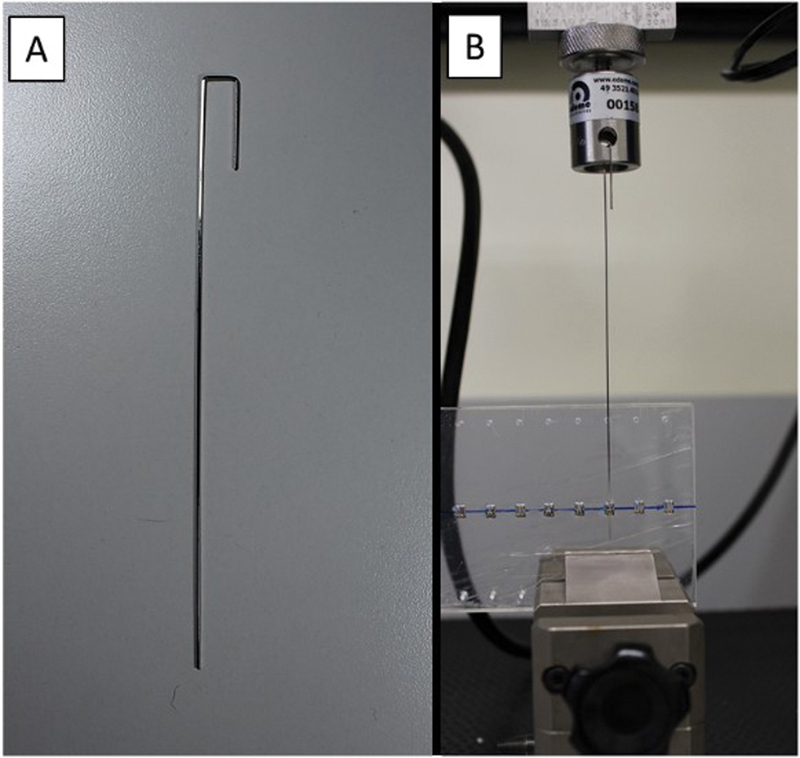
( **A** ) Wire shape used in the study. ( **B** ) Wire attached to the top of the universal testing machine.

The steel wires were made with a length of 16 cm, and in the final 3 cm on one side of the wire, two folds were made to fit it on the top of the universal testing machine ( Fig. 4 ).

#### Brackets Bonding

To avoid the presence of any material that could interfere with the results, the brackets and wires were cleaned with 70% ethyl alcohol. 6 14 16 Then they were submitted in a frictional movement for 10 seconds.

**Fig. 5:**
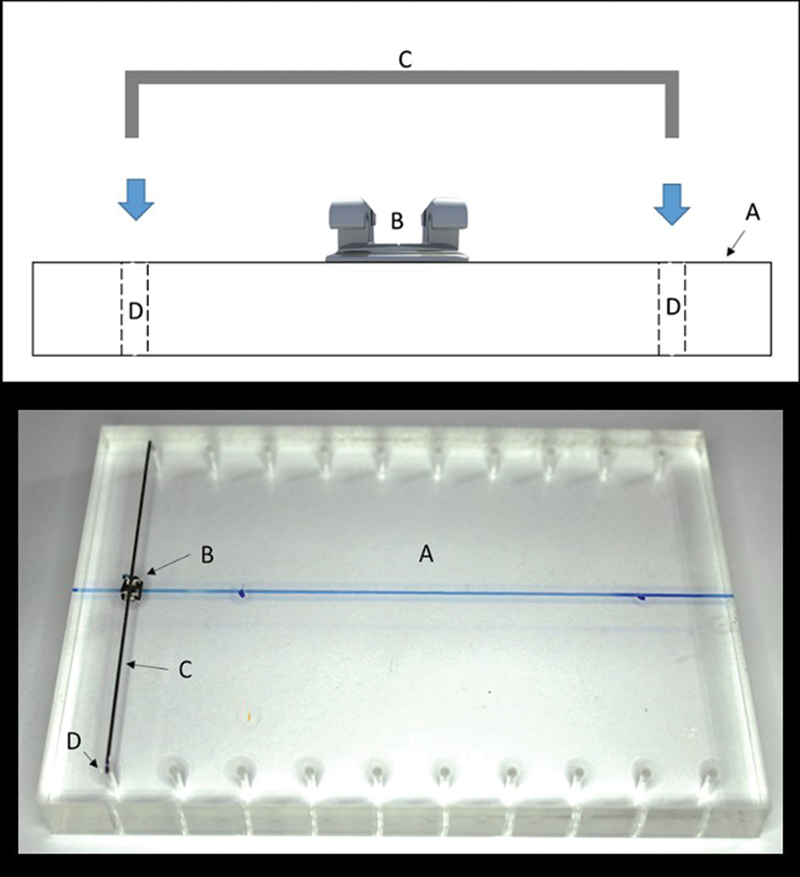
( **A** ) Acrylic device for bonding orthodontic brackets. ( **B** ) Orthodontic bracket bonded in the center. ( **C** ) Steel wire device 0.021 inch ×  0.025 inch in “ **U** ” shape. ( **D** ) Holes for inserting the “ **U** ” device and calibrating the orthodontic bracket bonding.

The brackets were bonded using cyanoacrylate ester-based instant glue (Super Bonder, Loctite Henkel, São Paulo, Brazil). 2 6 14 15 22 For standardization of positioning and bonding, the brackets were positioned parallel to the surface of the plate and bonded exactly in the center. As a guide for bonding, a device was made in rectangular 0.021 inch × 0.025 inch steel wire in a “ **U** ” shape, which was placed in the bracket slot, and its ends were fitted in the holes of the plate 5 to leave the wire entry angle equal to zero ( Fig. 5 ).

#### Confection of Metallic Ligatures

For the fabrication of the metal ligatures, 0.010-inch caliber wire threads, 15 4 cm long, were used. To standardize the format, the ligatures were made using plier-to-ligatures (Pliers Orthodontic 158; Ice, Cajamar, SP, Brazil). This same strapping pattern was used for the MLS and MLT groups.

#### Installation of Ligatures

To standardize the tests, all ligatures, of all groups, were exchanged after each test, except the self-ligating bracket, in which the bracket clip was opened, with a clinical dental probe, and closed, with a clinical dental clamp after each test. 23 An interval of 3 minutes between each test was established to release the initial tension of the ligature. 16 24

The H ligatures were installed manually and then tightened in the incise cervical direction of the bracket with Orthodontics Pliers Mathieu (Pliers Mathieu; Quinelato, Rio Claro, SP, Brazil) for HFM and with digital pressure for H3D. The same Orthodontics Pliers Mathieu was used to install the low-friction elastic ligatures (LT8). The conventional elastic ligatures were installed with an elastic ligature applicator instrument (Dental Morelli).

To remove the elastic ligatures (conventional and low friction) a dental probe was used. The same dental probe was used to remove the H ligature, where the tip was placed at the bottom of the ligature, lateral to the bracket, and the ligature was pulled down and out. This allows the H ligature to be stretched, making it easier to remove.

The metal ligatures were installed with the Orthodontics Pliers Mathieu, turning it clockwise, differing only in the number of turns (twists) performed. First, the number of turns was defined for the MLT group, which is the group in which the metal ligature was completely pressed, and for that, it required 10 complete turns. For the MLS group, six complete turns were agreed upon so that the ligature would be a little loose and allow the orthodontic wire to have more freedom of movement. 24 25

### Traction Test

For the simulation of the sliding mechanics, the static traction test was used in a straight line, with the orthodontic bracket remaining at rest about its base and with the wire sliding along the bracket slot. 6 10 14 15 17

To evaluate the frictional force in the studied systems, the universal testing machine EMIC DL 2000 (EMIC Test Equipment and Systems Ltda, São José dos Pinhais, Paraná, Brazil) was used to record the maximum force of each set. 12 15 26 A 5-N load cell was used 6 8 with a speed of 3 mm/min 16 26 for 2 minutes. 16 Each orthodontic bracket was tested five times 6 22 to find an average value for each bracket and, from that, an average for each group. The results obtained were transmitted to the computer connected to the testing machine and recorded in the Tesc software (Tesc; Intermetric, Mogi das Cruzes, SP, Brazil).

### Statistical Analysis

The statistical analysis of the data was performed using the Bioestat 5.3 software (Mamirauá Sustainable Development Institute, Tefé, Amazonas, Brazil). The Shapiro–Wilk test showed a non-normal distribution for the mean values of the groups ( *p* < 0.05). Therefore, statistical tests were performed to assess the existence of statistically significant differences between the groups through the Kruskal–Wallis test, followed by the Dunn test, and pairwise comparison ( *p* < 0.05; Table 2 ).

**Table 2:** Pairwise comparison: Dunn's test

		HFM	SLP	LT8	H3D	MLS	CEL	MLT
HFM	*p* -value	–	0.10	0.18	<0.01	<0.01	<0.01	<0.01
SLP	*p* -value		–	0.45	0.01	<0,01	<0.01	<0.01
LT8	*p* -value			–	0.09	<0.01	<0.01	<0.01
H3D	*p* -value				–	0.17	<0,01	0.01
MLS	*p* -value					–	0.17	<0,01
CEL	*p* -value						–	0.17
MLT	*p* -value							–

## Results

The average values followed by the respective friction standard deviations of the different ligation modes are shown in Table 3 .

**Table 3:** Mean values and standard deviation of the experimental groups (kgf)

Groups	HFM	SLP	LT8	H3D	MLS	CEL	MLT
Median and interquartile deviation	0.002 (± 0.000)	0.003 (± 0.001)	0.004 (± 0.000)	0.021 (± 0.008)	0.046 (± 0.015)	0.118 (± 0.011)	0.213 (± 0.019)
Statistical inference	A	A	A	B	C	D	E

Note: Different letters mean statistically significant differences, *p* < 0.05.

Abbreviations: CEL, conventional bracket with a conventional elastic ligature; LT8, conventional bracket with low friction elastic ligature; HFM and H3D, conventional bracket with H ligature; SLP, passive self-ligating bracket; MLS and MLT, conventional bracket with metallic ligature.

Comparing the different ligating modes, the three groups (HFM, SLP, and LT8) did not present a statistically significant difference between themselves, obtaining the lowest friction value among all groups. So the HFM ligature showed similar results to the SLP and less friction when compared with the H3D, MLS, CEL (control), and MLT groups. The MLT group showed statistically the highest friction value among all groups studied.

## Discussion

One of the basic principles of orthodontics is tooth movement, and this requires special care; and for this, light and continuous forces are indicated. The main factors that influence the force released to the teeth by the bracket/wire complex are wire thickness, wire deflection, ligation method, and frictional forces. 10 20 21 27 The frictional force in orthodontics is influenced by factors such as the material used, the size of the arch, and the ligature methods. 3 7 21 28 The present study evaluated the frictional force of a ligature designed by the authors (H ligature), with two different materials, in comparison to different available ligature modes.

Friction is defined as a tangential force that resists the movement of one surface against another, which acts in the opposite direction to the movement or tendency to the desired movement. 3 6 14 17 21 22 23 29 30 31 Although friction is not the only determining factor for treatment efficiency, it is crucial when used correctly and, at the same time, associated with the forces dissipated by the orthodontics arcs. 20 25 32 33

The straight-line vertical traction methodology of orthodontic wires connected to brackets by different ligature models is commonly used to compare the frictional force in different orthodontic systems. 6 10 14 15 17 The use of the universal testing machine is also supported by the literature since several studies evaluate friction in orthodontics using this test model. 6 14 15 16 17 22 24 32

The nickel-titanium (NiTi) alloy orthodontic wires, heat activated and by low caliber, are the most suitable for use together with the low-friction bracket/ligature in the initial stages of orthodontic treatment, as they have shape memory and allow the release of continuous forces without the need for frequent activations. 25 34 However, stainless steel orthodontic wires are still widely used and can be indicated, for example, for orthodontic traction and sliding mechanics. 17 22 24 32 For this reason, we used, in this research, 0.019 inch × 0.025 inch stainless steel orthodontic wires. 14 15 16 17 20 22

The first-choice orthodontic ligatures are currently the conventional elastic ligatures. 2 15 In this study, when comparing the friction of HFM with CEL, a significant statistical difference was found between them, with HFM showing the lowest friction values. The disadvantage concerning CEL is the longer working time for the H ligature, for its insertion and removal. The statistical comparison of HFM with the metallic ligatures revealed higher friction values for MLT followed by MLS. Such results are also due to the differences in the protocols for the installation of metallic ligatures, and these are not standardized. 2 14 17 25 This can be verified in a study where the metallic ligature was completely tightened on the orthodontic bracket, and after that, it was unrolled for three complete turns, leaving the loose ligature for the orthodontic wire to slide more easily, resulting in low levels of friction. 26 Another study showed that, like our results, when the metal ligature is completely tightened to the orthodontic bracket, the friction tends to be higher than even that of conventional elastomeric ligatures. 20

In contrast, self-ligating brackets have standardization in the installation of orthodontic wire and are easy to handle, 1 6 10 11 in addition to reducing friction when compared with traditional forms of ligation. 6 14 15 16 17 35 This fact can be confirmed in the present study because SLP showed statistically less friction in comparison to both conventional elastic ligatures and metallic ligatures. The SLP presented results similar to the HFM, but it has advantages such as promoting faster and more practical dental care since it does not require changing ligatures. 15 16 In this sense, the SLP is easier to handle than H ligatures. However, the most obvious advantage of HFM over the SLP is that it can be installed at any stage of the orthodontic treatment, especially when low friction is required. The H ligature can also be removed when more friction is needed, using another ligation method that promotes this effect, for example, traditional elastomeric ligatures. Another advantage of the H ligature concerning the self-ligating bracket is that it can be used with conventional brackets, which are relatively cheaper than the self-ligating brackets, thus reducing the cost of treatment. Another use would be for mechanics of traction of the anterior teeth, in which the ligature could be used only in the posterior region, to decrease the friction, whereas, in the anterior region, conventional forms of ligatures would be used.

Low-friction elastomeric ligatures are an alternative to self-ligating brackets. 13 This study used nonconventional elastomeric ligation with an “8” design (eight elastomeric ligature; Tecnident Orthodontic Equipment Ltd, São Carlos, SP, Brazil), whose studies demonstrate great similarity with the self-ligating ones, both showing low friction. 11 13 17 The results found in this work demonstrate the statistical similarity of LT8, SLP, and HFM. Although the time required to install and remove the LT8 is longer than the self-ligating bracket clips, removal was easier and faster than HFM.

The resin H ligature (H3D) showed intermediate values of friction force, resulting in statistically less friction than the conventional ligature groups (MLS, MLT, and CEL), and greater friction force than its metal version (HFM), as well as LT8 and SLP groups. These results show that H3D did not reach friction levels low enough to have the same effect as self-ligating ones, for example, to dissolve dental crowding or in orthodontic sliding mechanics. 5 17 19 20 23 28 The results also show that H3D does not hold the orthodontic wire sufficiently in the bracket slot to obtain good three-dimensional control, as is achieved with conventional ligatures. 19 In addition, the fragility of the material and the difficulty in handling it make the resin H ligature unsuitable for clinical use.

The development of H ligation sought to provide frictional forces close to those found with self-ligating brackets, with the aim that, clinically, similar benefits will be obtained. In this sense, HFM proved effective, revealing it as a new and promising device that can be used in situations where the professional needs to drastically reduce the friction in conventional brackets, also providing the possibility of exchanging for other types of ligatures, as soon as necessary. In this way, the orthodontist will have the possibility to customize the friction force caused for each specific case.

This study has some important limitations that need to be cited. The first one is that the tests were made using only brackets from one specific brand, and although H ligature could fit on other brand brackets such as the “Kirium Standard Metal Bracket” from 3M Abzil (3M, St. Paul, Minnesota, United States), more tests with other brands need to be performed. Besides that, the materials used to make the proposed ligatures do not seem to be ideal for use in everyday treatment, since the plastic version (H3D) proved to be very fragile, which makes its clinical use unfeasible, while the metallic version (HFM) is functional and resistant, but difficult to remove. As a result, to be used or even evaluated *in vivo* , new experiments are needed to select the ideal material, with adequate resilience and elasticity, similar to that found in elastomeric materials and NiTi alloys.

## Conclusion

- The lowest friction value was found for HFM, similar to SLP and LT8.
- The H3D ligature presented intermediate values of friction force concerning the other groups evaluated.
- The greatest frictional force was observed in the MLT group.

## Highlighted Manuscript

- The ideal orthodontic system appears to be one in which the friction levels can be switched, depending on the treatment phase, without changing brackets or increasing costs.
- The H ligature can be used for low friction or replaced by conventional ligatures to get high friction, depending on the need and the treatment phase.
- The new ligature is applied on conventional brackets, which are cheaper than self-ligating ones, bringing a similar friction level with probably lower cost.
- The new H ligature shows good friction levels, but other confection materials should be tested before having the final device.
